# Comparative evaluation of Nanopore polishing tools for microbial genome assembly and polishing strategies for downstream analysis

**DOI:** 10.1038/s41598-021-00178-w

**Published:** 2021-10-20

**Authors:** Jin Young Lee, Minyoung Kong, Jinjoo Oh, JinSoo Lim, Sung Hee Chung, Jung-Min Kim, Jae-Seok Kim, Ki-Hwan Kim, Jae-Chan Yoo, Woori Kwak

**Affiliations:** 1 JCBio. Co., Ltd., Seoul, 05836 Korea; 2 Department of Agricultural Biotechnology and Research Institute of Agriculture and Life Sciences, Seoul National University, Seoul, 05836 Korea; 3 Department of Laboratory Medicine, Kangdong Sacred Heart Hospital, Hallym University College of Medicine, Seoul, Korea; 4 Gencube Plus, Seoul, 08592 Korea; 5 Hoonygen, Seoul, 08592 Korea

**Keywords:** Bacterial genomics, Bioinformatics

## Abstract

Assembling high-quality microbial genomes using only cost-effective Nanopore long-read systems such as Flongle is important to accelerate research on the microbial genome and the most critical point for this is the polishing process. In this study, we performed an evaluation based on BUSCO and Prokka gene prediction in terms of microbial genome assembly for eight state-of-the-art Nanopore polishing tools and combinations available. In the evaluation of individual tools, Homopolish, PEPPER, and Medaka demonstrated better results than others. In combination polishing, the second round Homopolish, and the PEPPER × medaka combination also showed better results than others. However, individual tools and combinations have specific limitations on usage and results. Depending on the target organism and the purpose of the downstream research, it is confirmed that there remain some difficulties in perfectly replacing the hybrid polishing carried out by the addition of a short-read. Nevertheless, through continuous improvement of the protein pores, related base-calling algorithms, and polishing tools based on improved error models, a high-quality microbial genome can be achieved using only Nanopore reads without the production of additional short-read data. The polishing strategy proposed in this study is expected to provide useful information for assembling the microbial genome using only Nanopore reads depending on the target microorganism and the purpose of the research.

## Introduction

Microbial genomes present enormous differences in genomic structures, even among strains of the same species. These differences are caused by various reasons such as shorter generation time, higher mutation rate and evolution speed, and structural changes in genomes due to horizontal gene transfer (HGT)^1^. Thus, de novo assembly-based research that newly constructs the whole genome is more effective than resequencing-based research that reconstructs the genome by confirming differences based on the known genome of a specific microbial species. With the ongoing development of long-read sequencing technology, microbial genome assembly has already become the standard for microbial genome research methods.

The microbial genomic assembly has been invigorating since Oxford Nanopore Technologies commercialized its product Flongle, which uses replaceable small-size flow cells. The Flongle platform generates approximately 1 GB of sequencing data and its single flow cell is capable of generating over 100X coverage data, enough for most of microorganism genome assembly(< 10 MB). In addition, the cost of the experimental set-up of Flongle platform and its flow cell is cost-effective, so now many microbial genomic laboratories can easily equip their sequencing system and perform data generation for research. Such a shift in the research environment is expected to provide more insight into microbial genome evolution.

However, in the case of a microbial genome assembly using only long-reads such as Nanopore or Pacbio, there is a problem in that the accuracy of the base sequence produced is relatively lower than the short-read sequencing platforms such as Illumina^2^. Therefore, the polishing process to correct the remaining errors in the initial genome assembly is one of the most critical processes to achieve high-quality genome for downstream analysis. The hybrid method of assembly and polishing, which in addition uses a short-read sequencing platform to secure higher quality read information, is being widely used. Instead of using the long-read-only method, short-read sequencing can effectively deal with the remaining errors in the long-read assembly^2^. However, the use of additional short-read requires more financial costs for data generation and short-read sequencing devices, and also requires more time and manpower. For the laboratory and individual researchers, whose financial situation is relatively poor, the rising costs of sequencing can be a bottleneck for setting up an independent research environment from the data production to assembly for the downstream analysis. While it is possible to construct a high-quality microbial genome using only the long-read produced by Nanopore, is a very important issue in determining the paradigm of microbial genomic research.

In this situation, the enhancement and stabilization of the pore protein used in the Nanopore flow cell were in progress, and the base-calling algorithm that converts the generated electrical signals into sequence information was also advanced. Moreover, polishing tools that can demonstrate increased accuracy by using a variety of algorithms are continually being developed. Nanopolish^3^, a traditional Nanopore-based polishing tool, Racon^4^ and Medaka(https://github.com/nanoporetech/medaka), which are most frequently used as standards for Nanopore-based polishing, as well as various polishing tools such as Nextpolish^5^, PEPPER^6^, Apollo^7^, Homopolish^8^, and NeuralPolish^9^, recently developed in the last 1–2 years are available. These developments have enhanced the ability to construct a high-quality microbial genome using only Nanopore sequencing. However, the comparative evaluation of the newly updated Nanopore polishing tools and the possibility of producing a high-quality microbial genome assembly using only Nanopore reads have not yet been investigated.

In this study, using real genomics data of *E. coli*, one of the representative microorganisms, we evaluated the performance of various Nanopore-based polishing tools and verified the proper combination to construct the high-quality microbial genome assembly. In addition, we used a set of short-read data and hybrid polishing to compare and confirm if it was possible to construct a high-quality microbial genome assembly that could be used for downstream research by using only Nanopore-based polishing. We hope that this study will help researchers and laboratories studying microbial genomes obtain more independent research environments and provide useful information to establish appropriate analysis protocols for their own research purposes.

## Materials and methods

### Sample isolation

Multiplex PCR was performed for stool specimen with Seeplex Diarrhea-B2 ACE detection kit (Seegene, Seoul, Korea) to identify the presence of positive *E. coli* O157 strain for H7 and VTEC (verocytotoxin-producing *E. coli*) genes. The specimen was cultured with the MacConkey Agar with Sorbitol (BD, USA) and picked sorbitol-negative colorless colonies as presumptive *E. coli* O157:H7 strain for this study. Two probiotic species (*Lactococcus lactis* and *Streptococcus thermophilus*) were provided by CTCBio. Inc. Seoul, South Korea.

### Genome sequencing, QC & assembly

Data generation was conducted using three sequencing platforms, Oxford Nanopore Minion, Flongle, and Illumina Miseq. For Nanopore sequencing, SQK-LSK109, NBD-114, FLO-MIN106(R 9.4), and FLO-FLG001 were used for library construction and data generation. For Illumina sequencing, Miseq reagent kit V2 and Nextera DNA Flex Library Prep were used for library construction. All data generation process was conducted by following the protocols from the manufacturer.

Base-calling and demultiplexing were conducted using Guppy v5.0.7^10^. Guppy_basecaller was used with the dna_r9.4.1_450bps_hac model and guppy_barcoder was used with parameter –barcode_kits NBD-114. After base-calling and demultiplexing, the sequencing artifact was removed using Porechop (https://github.com/rrwick/Porechop) for assembly. An additional version of Porechop trimming data was generated for Nanopolish with –discard_middle parameter because it did not allow the split read. For Illumina Miseq data, sequencing artifact was removed using Trimmomatic^11^ with ILLUMINACLIP:TruSeq3-PE.fa:2:30:10:2:keepBothReads parameter.

Initial genome assembly was conducted using CANU^12^ with genomesize = 4.8 m parameter. CANU result provides circular information for the assembly, the raw assembled sequence was trimmed using suggested circular information from CANU. In the case of two probiotic species, CANU could not generate circularized assembly, and additional circularization was conducted using Circulator^13^.

### Genome polishing and assessment

8 polishing tools (Racon v1.4.21, Medaka v1.3.3, PEPPER v0.1.5, Homopolish v0.2.1, Nextpolish v1.3.1, NeuralPolish 2021–05-21, Nanopolish v0.13.3, and Apollo v2.0), which can conduct polishing using only nanopore reads, were used for this study. Reads were mapped to initial assembly using Minimap2^14^ v2.17-r941 with -ax map-ont parameter and sorted using Samtools^15^ v1.10. Most of the polishing tools used in this study were conducted with default parameter and additional parameter setting was used only for some polishing tools. Racon was used for additional polishing with parameters adjustment because there is a well-known parameter suggestion for the Racon-Medaka polishing combination. For the Racon-Medaka combination, Racon parameter was adjusted to -m 8 -x -6 -g -8 -w 500. For PEPPER polishing, PromethION_r941_guppy305_HAC_microbial.pkl model was used which is fitted to Minion pore version used in this study. For Homopolish, -m R9.4.pkl parameter was used. For short-read-based polishing, short-reads from Miseq were mapped to initial assembly using bwa-mem2 v2.1, and polishing was conducted using Pilon^16^ v1.23 with the default parameter.

For polished genome evaluation, BUSCO^17^ v5.1.1 was used with enterobacterales_odb10 database. Gene contents were identified using Prokka^18^ v1.14.6, and gene order and structure were compared, manually. Pseudogene count was calculated using prokka-suggest_pseudogenes.pl script. Read mapping was visualized using IGV^19^ v2.9.0.

## Results

### Data generation and assembly

Generated data from two sequencing platforms were shown in Table 1. CANU assembly for initial draft genome successfully constructed whole circular chromosome and one circular plasmid. Information of constructed genome and plasmid is summarized in Table 2.

**Table 1:** Summary of generated sequencing data for *E. coli* genome used in this study.

Library name	Sequencing platform	Read type	Read count	Bases (bp)
Short-read	Illumina Miseq	Paired-end	2,302,658	341,783,000
Long-read	Nanopore Minion	Single-end	2,350,791	10,392,655,168

**Table 2:** Summary statistic of the circularized initial *E. coli* genome assembly from CANU.

Chromosome		Plasmid	
Number of sequences	1	Number of sequences	1
Number of A's	1,356,990 (24.72%)	Number of A's	23,515 (25.49%)
Number of C's	1,389,721 (25.32%)	Number of C's	23,810 (25.81%)
Number of G's	1,387,128 (25.27%)	Number of G's	20,254 (21.96%)
Number of T's	1,355,108 (24.69%)	Number of T's	24,671 (26.74%)
Total	5,488,947	Total	92,250

### Evaluation of single tool polishing

**Figure 1:**
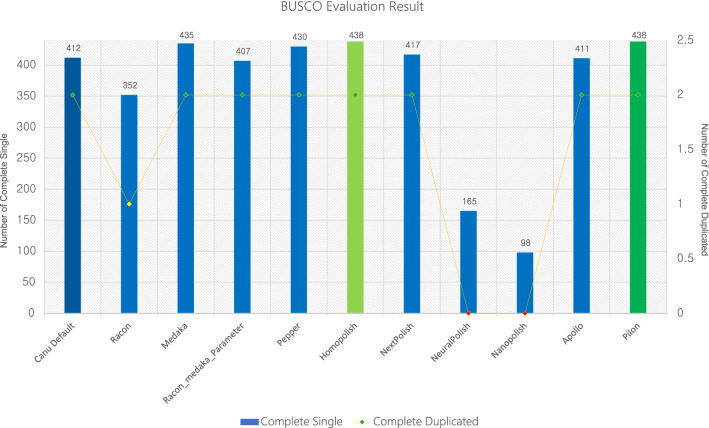
BUSCO evaluation result using enterobacterales_odb10 for each polishing tool. Bar indicates the number of single complete BUSCO genes and yellow line indicates the number of duplicated complete BUSCO genes.

**Figure 2:**
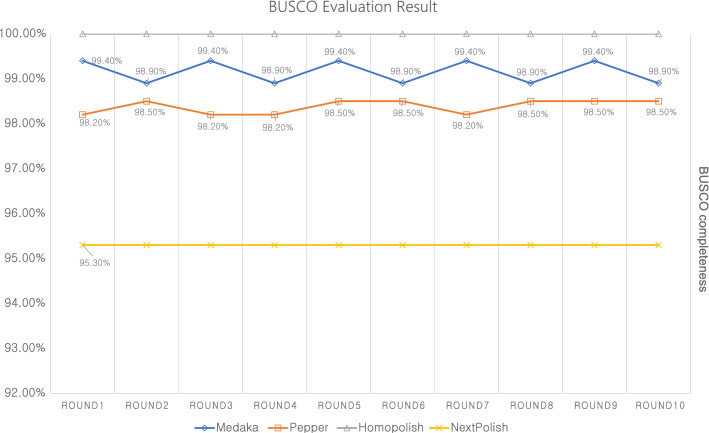
BUSCO evaluation result of 10 round iterative polishing for 4 polishing tools.

**Figure 3:**
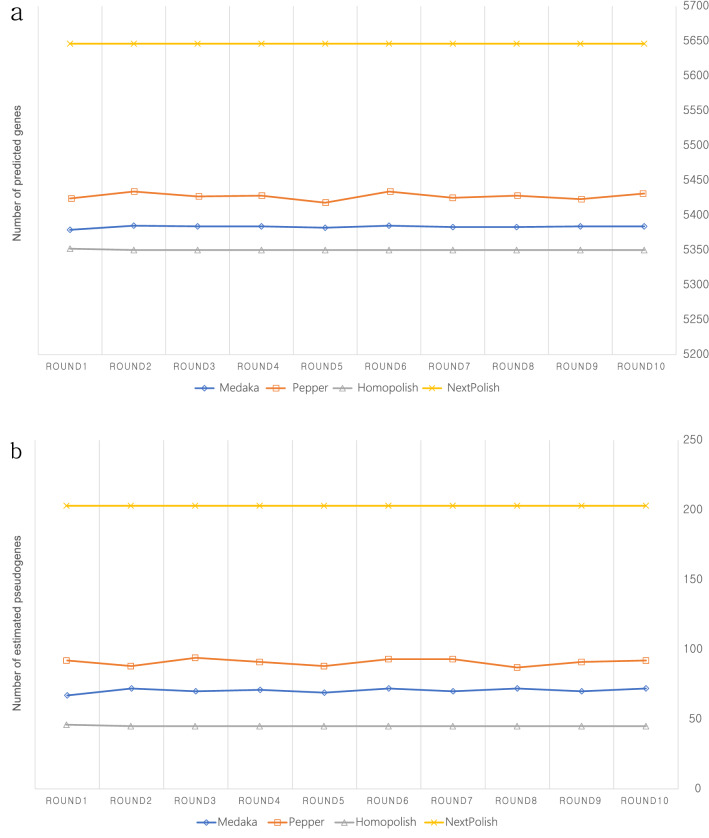
Prokka gene prediction result of 10 round iterative polishing for 4 polishing tools. (**a**) the number of predicted genes in each round, (**b**) the number of estimated pseudogenes in each round.

Table 3 shows the polishing tools used for evaluation in this study and Fig. 1 shows the BUSCO evaluation result for each polishing tool. In the enterobacterales_odb10 used for BUSCO evaluation in this study, there are 440 BUSCO genes. Based on the pilon polishing with Miseq short-reads, there are 2 duplicated BUSCO genes in the *E. coli* genome used in this study. Raw assembly from CANU showed 412 single copies and 2 duplicated BUSCO genes, and the completeness was 94.1%. 23 genes were fragmented and 3 genes were missing. Polishing tools should improve the accuracy and quality of the assembled genome, but some polishing tools have shown worse results than the initial assembly in BUSCO evaluation. In terms of BUSCO evaluation, Racon (default and medaka parameter), NeuralPolish, Nanopolish, and Apollo could not improve the quality of assembled genome. Medaka, PEPPER, Homopolish, and NextPolish showed the improved BUSCO evaluation result compared to the initial assembly. Among them, only Homopolish(100% completeness with 2 duplicated BUSCO) showed the same result with Pilon polishing using Miseq short-reads. In addition, we tested 10 round iterative polishing for 4 polishing tools which showed better results than initial assembly. The purpose of this test goal is to assess wheter iterative polishing can increase accuracy. Figure 2 shows 10 round iterative polishing results for 4 polishing tools in BUSCO evaluation. Two polishing tools (Homopolish and Nextpolish) showed no change in BUSCO evaluation result at each iteration and the other two polishing tools (Medaka and PEPPER) showed fluctuating results in BUSCO completeness. Figure 3 shows gene prediction results using Prokka for 10 rounds of genome polishing using 4 polishing tools. Nextpolish did not show the different results in each polishing round but showed the largest number of estimated pseudo-genes compared with other polishing tools. Homopolish showed almost the same result in every polishing round and that was the most similar result with short-read-based polishing using Pilon. In the case of Medaka and PEPPER, they showed a similar fluctuation pattern in the gene prediction result like BUSCO evaluation. Each iterative polishing using 4 tools showed the same result in the rRNA (22) and tRNA (102), and it was the same with the prediction result from the short-read-based polishing approach using Pilon.

**Table 3:** List of the polishing tools used in this study.

Tools	Authors	Published Year
Nanopolish^3^	Nicholas J Loman	2015
Racon^4^	Robert Vaser et al	2017
Medaka	Oxford Nanopore	2018
NextPolish^5^	Jiang Hu et al	2019
PEPPER^6^	Kishwar Shafin et al	2020
Apollo^7^	Can Firtina et al	2020
Homopolish^8^	Yao-Ting Huang et al	2021
NeuralPolish^9^	Neng Huang et al	2021

### Evaluation of combination polishing

**Figure 4:**
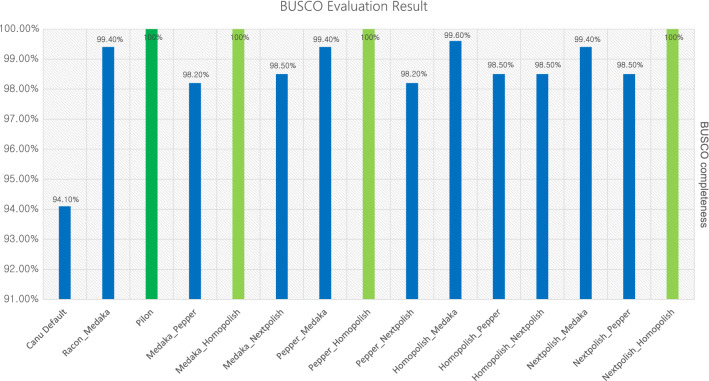
BUSCO evaluation result for combination polishing using enterobacterales_odb10. Bar indicates the completeness of BUSCO. Green color indicates the result of short-read based pilon polishing. Light green color indicates the highest accuracy from Nanopore read based polishing.

**Figure 5:**
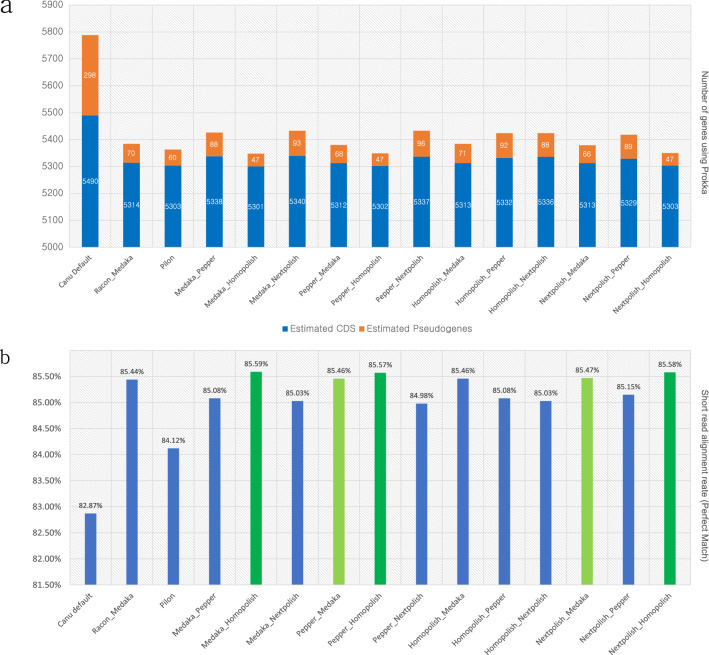
Gene prediction result using Prokka and exact read alignment rate using bowtie2 for each polishing combination. (**a**) Number of predicted genes and estimated pseudogenes using Prokka. (**b**) Alignment rate of perfect matched Illumina short-reads to the polished genome.

Each polishing tool uses its own specific algorithm to improve the accuracy of assembly. Therefore, we tested every combination of 4 polishing tools which showed a better result than the initial assembly. And we also carried out an additional well known polishing combination Racon and Medaka for Nanopore long-read. Figure 4 and Fig. 5 shows the evaluation and prediction result of BUSCO and Prokka, respectively. In BUSCO analysis, the second-round polishing with Homopolish showed 100% completeness regardless of the previous polishing tools. In the case of Racon × Medaka combination, there was no difference in the BUSCO result (99.4% completeness) compared to single Medaka polishing. Among combinations except for second-round Homopolish, Homopolish × Medaka combination showed the highest BUSCO completeness (99.6%). Figure 5a shows the result of polishing combination effectively reduced the estimated pseudogenes and false positively predicted genes in the initial assembly. Same as BUSCO analysis, the second round with Homopolish showed the most similar result with the Pilon polishing in Prokka gene prediction and the estimated number of pseudogenes was the same regardless of previous polishing tools. Except for the second round Homopolish combination, PEPPER × medaka, and Nextpolish × Medaka showed similar gene prediction result with Pilon polishing. Figure 5b shows the perfect read alignment rate of Illumina short-read to the assembly using each polishing combination. Medaka × Homopolish showed the highest alignment rate (85.59%) and Nextpolish × Medaka showed the highest alignment rate (95.47%) among read-based polishing. As the exact alignment rate increased, the number of estimated pseudogenes also tended to decrease.

### Comparison of gene order and structure

**Figure 6:**
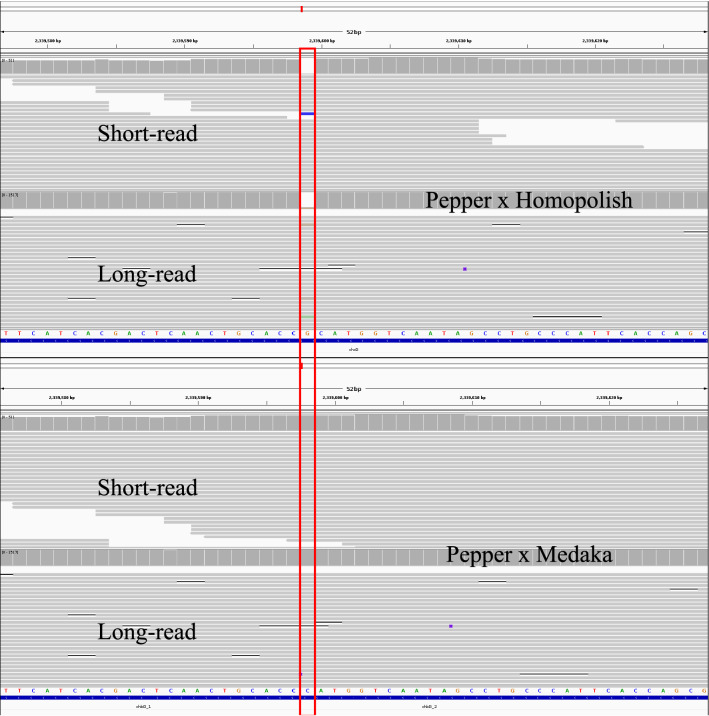
Visualized read alignment to the polished genome using IGV. Polished genome using Homopolish did not contain the sample-specific variation, however, read-based polishing PEPPER × Medaka polishing successfully reflect the sample-specific variation.

Gene orders from 6 different polished assemblies (Pilon, Medaka × Homopolish, PEPPER × Homopolish, Nextpolish × Homopolish, Racon × Medaka and PEPPER × Medaka) were compared to identify the detailed gene prediction result with the short-read based polishing using Pilon. Table 4 summarizes of the difference and types between combined polishing and short-read-based Pilon polishing. PEPPER × Homopolish combination showed the lowest number of differences compared to short-read-based Pilon polishing. In addition, PEPPER × Homopolish and Racon × Medaka combination showed no missing prediction compared to short-read Pilon polishing. Overall, the second round Homopolish combination showed lower numbers of differences compared to a read-based polishing combination such as Racon × medaka and PEPPER × medaka, and there was a tendency of mismatch type in polishing combination. For the combination using the second round Homopolish, most of the mismatches were found to be merged. However, Racon × Medaka and PEPPER × Medaka Combination, which is finished using the produced read, the gene is split compared to the polishing combination finished with Homopoish. To verify the difference that occurs when short-read is used, the results of PEPPER × Homopolish were additionally polished with Pilon, and the two results were compared. The results are shown in Table 5. Due to performing additional polishing using short-read, there was a difference in the predictions of 18 (approximately 0.3% of total) of the total 5475 features. Except for 7 hypothetical proteins, there were differences in a total of 11 genes, 4 of which were merged or split, and the remaining 7 had differences in the length of CDS. Figure 6 shows why this difference occurs. Homopolish shows outstanding polishing results in most cases, but if the mutation included in the genome is strain-specific or if the variant is not dominant among homologous sequences used in Homopolish, it can be missed. In the case of the genome polished with Homopolish, as shown in Fig. 6, one chbG gene seems to exist. However, as can be seen from the mapping coverage of the produced read, in the case of the *E.coli* genome used in this study, the presence of Indel at the position of 2,339,591 bp can be checked in both long- and short-read. This result came from 20 homologous genomes used in Homopolish. Among 20 homologous genomes used by Homopolish, only one genome contains this specific variant, and the consensus process using these genomes made the false correction. Consequently, when we conducted read-based polishing, the specific mutation information of the target microorganism can be properly reflected. This is a limitation of Homopolish, which performs polishing based soley on known genomic information without using the read data generated from the sample.

**Table 4:** Summary of the number of differences and types in Prokka predicted genes in combination polishing compared to short-read pilon polishing.

Combination	Total Mismatch	Gene Merged	Gene Split	Additional Prediction	Loss Prediction
Medaka × Homo	26	18	1	5	2
PEPPER × Homo	24	19	2	3	0
Next × Homo	28	20	0	6	2
Racon × Medaka	54	16	33	5	0
PEPPER × Medaka	50	15	28	5	2

**Table 5:** Differences between PEPPER × Homopolish and PEPPER × Homopolish × Pilon.

Position	Type	Description	Difference
2,229,653–2,231,971	CDS	Ion-translocating oxidoreductase complex subunit C	Split
2,276,362–2,276,416	CDS	putative oxidoreductase YdhV	Merged
2,339,610–2,339,601	CDS	Chitooligosaccharide deacetylase ChbG	Merged
4,057,083–4,057,490	CDS	putative fimbrial-like protein YraK	Missing
2,518,544–2,517,555	CDS	Tyrosine recombinase XerC	195 bp
2,881,870–2,882,727	CDS	Nickel/cobalt efflux system RcnA	−6 bp
3,112,312–3,111,914	CDS	L-rhamnonate dehydratase	72 bp
3,113,114–3,112,350	CDS	L-rhamnonate dehydratase	21 bp
4,169,438–4,170,403	CDS	tRNA-dihydrouridine synthase B	−105 bp
5,017,871–5,018,644	CDS	Acetylglutamate kinase	3 bp
5,061,026–5,061,718	CDS	NADH pyrophosphatase	81 bp

7 predicted hypothetical proteins, not match between two polished assemblies are not listed.

### Application of polishing combination to 2 model microbes

**Figure 7:**
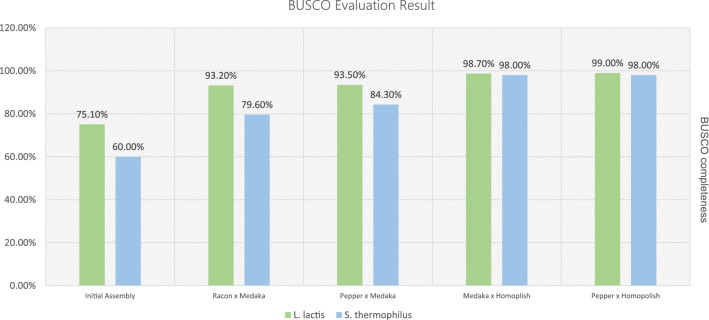
BUSCO evaluation result using enterobacterales_odb10 for two probiotic species.

To evaluate the polishing combination, which shows comparatively better performance than others in *E. coli* genome, four polishing strategies (Racon × Medaka, Pepper × Medaka, Medaka × Homopolish and Pepper × Homopolish) were applied to two additional model microbes (*Lactococcus lactis* and *Streptococcus thermophilus*). Table 6 shows the summary statistics of generated Nanopore read and Table 7 shows the summary information of constructed initial assembly for two species. Figure 7 shows the BUSCO evaluation result of two microbes for each polishing combination strategy. Result of BUSCO evaluation of the initial assembly were low (75.1% and 60.0% for *L. lactis* and *S. thermophilus*, respectively) but the applied four combination polishing did improve the BUSCO completeness. Among them, similar to the result observed in the *E. coli* genome, the highest completeness result was observed in the combination finished with Homopolish. Pepper × Homopolish combination shows slightly better performance than Medaka × Homopolish combination (99.0% for *L. lactis* and 98.0% for *S. thermophilus,* respectfully). Among the non-reference-based combinations that did not use Homopolish, the Pepper × Medaka combination showed relatively high completeness compared to the Racon × Medaka combination in *L. lactis* genome. Although short-read data for these two species have not been used, almost complete BUSCO results can be obtained by using only Nanopore read with an appropriate polishing combination.

**Table 6:** Summary of generated sequencing data for *L. latics and S. thermophilus* genome used in this study.

Library name	Sequencing platform	Read type	Read count	Bases
L_lactis	Nanopore Flongle	Single-end	212,379	846,604,405
S_thermo	Nanopore Flongle	Single-end	153,886	1,228,787,058

**Table 7:** Summary statistic of the *L. lactis* and *S. thermophilus* genomes after circularization using Circlator.

*L. lactis*		*S. thermophilus*	
Number of sequences	1	Number of sequences	1
Number of A's	807,167 (32.31%)	Number of A's	560,550(30.25%)
Number of C's	440,839 (17.65%)	Number of C's	362,323(19.55%)
Number of G's	444,943 (17.81%)	Number of G's	361,434(19.50%)
Number of T's	804,929 (32.22%)	Number of T's	569,043(30.70%)
Total	2,497,878	Total	1,853,350

## Discussion

### Strategies for microbial genome polishing using only Nanopore Reads

The accuracy of Nanopore sequencing increases with the ongoing development of Nanopore pore protein and its base-calling algorithm. This leads to the growing possibility of securing the high-quality microbial genome without additional production of short-read. Moreover, as polishing tools which can be carried out using only Nanopore are being developed and updated, the quality-enhancing potential is growing.

According to the result of this study, the choice of an appropriate polishing tool is the most important for the high-quality genome assembly using only Nanopore since there is a significant difference in the performance of each tool algorithm. This study also confirmed that even a relatively new polishing tool, which should have an algorithm that compensates for the disadvantages of the existing tool, does not always show better results.

In particular, each polishing tool has shown numerous differences in the process of data usage and processing time that lead to a great difference in accessibility and applicability. For example, an older tool such as Nanopolish requires the use of the Fast5 file, and the corresponding information and the Fastq file must be matched. Therefore, if the adapter trimming is performed using Porechop, the dataset from which the read information split by the middle adapter had to be removed, and additional dataset must be configured separately. The polishing process also took an incomparable amount of time compared to other tools with good performance, but the results were not satisfactory. Similarly, relatively newly developed Apollo, although it is not an old tool like Nanopolish, only Fasta files with a constant number of lines are used as input. Not only does the process take more steps like converting the Fastq file to a specific Fasta form, but also takes much more processing time than other tools and has not shown polishing improvement.

Among the polishing tools used in this study, it was confirmed that the result using Homopolish, PEPPER, and Medaka could produce the quality most similar to the result of using short-read in addition. In the case of Homopolish, the accuracy was the highest, but the polishing process is carried out with a known reference genome rather than the read produced. Since *E. coli* used in this study was one of the species with the availability of high-quality open genomes, the result of the Homopolish showed better. If there is no high-quality genome available or if the number is inadequate, it is difficult to expect the same return. For this reason, with Homopolish, there is a limitation that can be used where there are enough high-quality genomes available in the NCBI database. As Homopolish does not use produced read information, the side effect of recovering pseudogenized or damaged genes due to a strain-specific mutation during the polishing process is also occurs in small proportions.

When using the produced reads, the combination of PEPPER and Medaka produced the least mismatching result when compared to Pilon using short-read. In the case of PEPPER, an error profile model that can occur in a specific pore version is additionally used. Updating the error profile will further improve its accuracy, and if the model is continuously updated based on the pore version change, more accurate genomes can be achieved.

Based on the results of this study, a microbial assembly strategy using Nanopore alone is recommended as follows. If the target species is a well-known species and there are numerous high-quality open genomes available in the NCBI database, the combination of PEPPER × Homoplish or Medaka × Homopolish can be suggested. For a researcher intensively studying a previously unknown specific microbial species with no related high-quality open genome, but if there is a high-quality genome derived from a hybrid method, building a custom database and using it for Homopolish can be a strategy to construct a proper microbial genome. For the gene of major interest, performing the manual curation based on mutation information by long-read mapping and Medaka can complement some cases of Homopolish that do not reflect specific individual mutations and help with follow-up research. If the target species of the research is not well known or if there are not many high-quality open genomes available, it is preferable to use the read-based polishing, PEPPER × Medaka combination. This combination of read-based polishing tool, which can be used when Homopolish is not available, shows the most similar results of using extra short-read in microbial genome assembly compared to others.

### Can Nanopore solely construct high-quality microbial genome?

‘Is it possible to construct a high-quality microbial genome assembly that will be used for further analysis by using Nanopore alone?’ As noted previously, the answer to the question may vary depending on the type of microorganism to be studied and the purpose of the study. By comparing a polished assembly using only Nanopore with a polished assembly using additional short-read, only a very small number of genes showed a difference in the gene content. Most of the genes showing the difference were either hypothetical or putative proteins, or merge or split of the same genes. Therefore, even by microbial genome assembly using only Nanopore, it is possible to construct a genome assembly of sufficient quality to fully understand the genetic content of the corresponding microorganism. However, since Homopolish based on high-quality genomes is essential for this result, high-quality genome information needs to be secured through the production of additional short-read data based on the availability of the target microbial species to be studied. Moreover, during the discovery of microscopic mutations such as phenotype differences due to single point mutation or differences in the structure and sequence of strain-specific genes, additional short-read production is advised. Offsetting the weakness of using only long-read will reduce the false positive of constructed genome-based research outcomes.

Even for target microbial species that cannot use Homopolish, the general characteristics of the microbial genome can be understood by combined polishing such as PEPPER × Medaka. Although the short-read is not necessarily produced in addition, depending on the objective of the research, it is still possible to secure a sufficient level of assembly necessary for further research by using only Nanopore. Furthermore, thanks to the continuous development of pores and the associated algorithms, the accuracy of Nanopore sequence analysis is being improved. Shortly, we expect that a high-quality microbial genome to be produced using only Nanopore, regardless of the research objective and the target microorganism.

## Data Availability

All generated sequencing data in this study can be found in the NCBI accession PRJNA759000 and all polished genomes in this study can be found in https://github.com/asleofn/Polishing.
